# SIRAC: Supervised Identification of Regions of Aberration in aCGH datasets

**DOI:** 10.1186/1471-2105-8-422

**Published:** 2007-10-30

**Authors:** Carmen Lai, Hugo M Horlings, Marc J van de Vijver, Eric H van Beers, Petra M Nederlof, Lodewyk FA Wessels, Marcel JT Reinders

**Affiliations:** 1 Bioinformatics group, Delft University, Delft, The Netherlands; 2 The Netherlands Cancer Institute, Amsterdam, The Netherlands

## Abstract

**Background:**

Array comparative genome hybridization (aCGH) provides information about genomic aberrations. Alterations in the DNA copy number may cause the cell to malfunction, leading to cancer. Therefore, the identification of DNA amplifications or deletions across tumors may reveal key genes involved in cancer and improve our understanding of the underlying biological processes associated with the disease.

**Results:**

We propose a supervised algorithm for the analysis of aCGH data and the identification of regions of chromosomal alteration (SIRAC). We first determine the DNA-probes that are important to distinguish the classes of interest, and then evaluate in a systematic and robust scheme if these relevant DNA-probes are closely located, i.e. form a region of amplification/deletion. SIRAC does not need any preprocessing of the aCGH datasets, and requires only few, intuitive parameters.

**Conclusion:**

We illustrate the features of the algorithm with the use of a simple artificial dataset. The results on two breast cancer datasets show promising outcomes that are in agreement with previous findings, but SIRAC better pinpoints the dissimilarities between the classes of interest.

## Background

Genomic alterations in DNA copy number are important events in cancer development [1]. A tumor suppressor gene can be disabled by the physical loss of the gene, or similarly an oncogene may be over-expressed via the amplification of the region where it is located. The identification of chromosomal aberrations is, therefore, a powerful instrument in studies of cancer. It may suggest target genes for new drugs or shed light on the mechanisms which regulate the response to therapies [2-4].

The first approach to search for copy number alterations in CGH has been made by Kallioniemi *et al. *[5] using metaphase chromosomes. The extensions of this technique employ array technology to perform a high resolution scan of the genome. As reviewed by Pinkel *et al. *[3], several array CGH (aCGH) techniques have been developed. The spotting technology makes use of BAC clones (100 – 200 kb), cDNA clones (~100 – 1000 bp) and lately oligonucleotides (30 – 100 bp). More recently, *in-situ *technologies synthesize small oligonucleotides directly onto the array. Since the oligos can be a few tens bp long, higher resolution are reached, if a good coverage of the genome is adopted.

An important challenge to analyze aCGH data is to find the aberrated chromosomal regions specific to the problem under study, e.g. to distinguish between subtypes of cancer. In order to reach this goal, three groups of approaches can be found in the literature. The first group of approaches uses only the aCGH data. First they identify the amplifications/deletions in each sample individually, and then search for the common aberrations between the samples. The identification per sample of chromosomal regions of aberration is a task in itself that has been approached in several ways. The simplest solution is the application of a threshold. The DNA-probes (BAC clones, cDNA clones or oligonucleotides) which exceed the threshold are considered amplified/deleted [6-9]. The choice of the threshold is a very critical parameter. Moreover, the threshold methods have the limitation that they do not take into account the spatial location of the DNA-probes. Since amplicons (i.e. regions that are amplified in a sample) are commonly assumed to involve more than a single DNA-probe, the spatial position is an important factor. Several more complex algorithms have been developed to identify, per sample, the aberrated regions in more robust ways. Lai *et al. *[10] reviewed eleven different methods available in the literature. Numerous segmentation methods have been proposed to divide the aCGH profile in piece-wise constant segments, and a likelihood function is used to estimate the model parameters from the data. For example, Picard *et al. *[11] modeled the aCGH profile with a random Gaussian process and introduced an adaptive penalized likelihood to estimate the segments and their locations. Jong *et al. *[12,13] proposed a genetic algorithm to maximize the likelihood function. A different approach was introduced by Wang *et al. *[14]. They identified the regions of amplification/deletion via a hierarchical clustering along the chromosome.

The biologically relevant aberrations are not the ones that characterize a single sample, since these can be the consequence of the genomic instability of the particular tumor. The more interesting aberrations are the ones shared by many samples, ideally by all the samples in the same class. Previous studies combined the information of the per sample aberration by looking at the frequency of patients that carry the aberration [6,8,14-17]. Again a threshold on the minimal frequency is chosen. For example, Fridlyand *et al. *[15] require the aberrations to be present in more than 50% of one class and less than 30% of the second class, whereas Hyman *et al. *[16] demands that the aberration be present in at least two specimens. These approaches have in common that the class information is taken into account only in the second stage of the analysis, i.e. when computing the aberration frequency across the samples. In the first phase also the aberrations common to more classes are considered, even if they are not of interest for the study. This introduces an extra parameter when evaluating the significance of the aberrations to distinguish the classes of interest. Recently, Diskin *et al. *[18] proposed a more complex and systematic way to evaluate the significance of aberrations across samples. However, they require the input data to be discretized per sample into amplifications and deletions. This step can be performed using one of the mentioned above methods, but makes the results dependent on the particular approach chosen for discretization.

A second group of approaches to detect aberrations across samples uses only the gene expression data together with the chromosomal location of the genes. The assumption is that an amplification directly affects the expression of the genes. Therefore, the genes in that region should have a detectable common over-expression. Similarly, the genes located in a deletion would have a detectable under-expression. Furge *et al. *[19] applied the binomial test per sample on the genes within a given window size. In order to cover the whole genome, the window is slid across the genome, performing a test at fixed intervals. The z-scores of the test for a particular location are averaged across several window sizes and a threshold is chosen. The locations above/below the threshold are identified as regions of chromosomal aberration. Levin *et al. *[20] applied a Poisson model to the expression data and incorporated the genomic location in their model-based scan statistic. These results are compared per sample with the aCGH data. Yi *et al. *[21] used a sliding window size of 5 genes to test the significance of the region according to two scores, which account for the homogeneity of behavior in the window and the power of the genes in discriminating the classes of interest. Dressman *et al. *[22] observed that the genes over-expressed shared the same location, hypothesized an amplification and validated their findings with PCR. These studies show interesting examples of aberrations identified using the transcriptome data only. However, the assumed strong correlation of aCGH and expression could not be detected by other studies [17,23-25]. Since the alteration in expression may be due to diverse mechanisms, the potentially underlying chromosomal aberrations would need to be verified either by PCR or FISH, if the number of loci to be tested is tractable, otherwise by aCGH data. The advantage of the aCGH technology arises in the genome-wide coverage of the analysis.

The third group of approaches combines aCGH and expression data to detect regions of chromosomal aberration. The SLAM algorithm (Adler *et al. *[26]) is a prime example of this group. First the SAM analysis [27] is applied to the aCGH data in order to identify the DNA-probes which distinguish the two classes. Then the focus is on the DNA-probes that are correlated with the expression data. Based on the observation that many of them were on the same chromosome arm, the hyper-geometric distribution was used to test the significance of that arm.

Inspired by the work of Adler *et al. *[26], we propose a supervised procedure to identify chromosomal regions of aberration using solely aCGH data. We use the SAM analysis to determine the "relevant" DNA-probes, i.e. the DNA-probes that distinguish the classes of interest. While Adler *et al. *[26] evaluated only a single location chosen in an ad hoc fashion, we build a systematic search to test the whole genome. We adopt a sliding window approach similar to the one proposed by Furge *et al. *[19]. More specifically, we apply a hyper-geometric test to window sizes of different length, and test the significance of the number of relevant DNA-probes in those windows. Our algorithm belongs to the first group of approaches, since it uses only aCGH data. However, it differs from the typical approaches in this group in the following ways. First of all it focuses only on the aberrations specific to the problem of interest, by exploiting the class labels in the first step (recognizing relevant DNA-probes). Importantly, no discretization, smoothing or segmentation algorithms are applied to the aCGH data. This leads to the advantage that the data is not altered based on the preconceived models that these algorithms presume. Moreover, we also avoid the optimization of the parameters that these models usually require (avoiding results sensitive to these choices). The use of the hyper-geometric test corrects for the non-uniform background distribution of the DNA-probes. This is particularly important since the DNA-probes are not equally spaced along the genome. In this way we build a robust algorithm to identify areas of interest specific to the problem under study. We illustrate the benefit of our procedure on an artificial dataset, and show the results on two breast cancer datasets.

### Algorithm description

**Figure 1:**
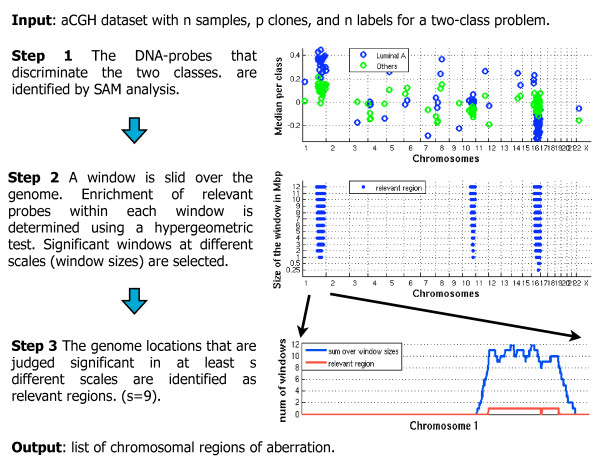
**Description of the SIRAC algorithm**. Illustration of the algorithmic steps of SIRAC. The corresponding results for the *NKI *dataset are shown. The data is labeled according to the cancer subtypes introduced by Sorlie and Perou [30, 31, 32]; in this example the label Luminal A subtype versus all others subtypes is used. In Step 1 the relevant DNA-probes are selected. Each DNA-probe is plotted on the genomic location with two circles of different color representing the median value of the samples in the two classes. In Step 2, the vertical axis represents the different window sizes, the blue lines along the genome (the horizontal axis) show the regions judged significant by the algorithm. In the final step, Step 3, the number of window sizes for which the location is judged significant by the hyper-geometric test are shown along the vertical axis. The relevant region selected when *s *= 9 is highlight by the red curve.

Figure 1 illustrates our algorithm SIRAC (Supervised Identification of Regions of Aberration in aCGH data). A detailed description is given in Appendix 1. An aCGH dataset *D *and its label set *y *provide the starting point. The procedure consists of three steps.

STEP 1. We identify with the SAM analysis [27] the DNA-probes which discriminate between the classes of interest. We call these DNA-probes the "relevant" probes. In Figure 1 (Step 1) the relevant DNA-probes are depicted on the genomic location. Each probe is plotted with two circles of different color representing the median value of the samples in the two classes.

STEP 2. We test, in a systematic way, whether the number of relevant DNA-probes in a region is higher than expected by chance. For this purpose we use the hyper-geometric test for a genomic position, and test whether the fraction of relevant DNA-probes in the window of length 2*w *represents a significant enrichment. By sliding the window of observation along the genome, shifting it a single DNA-probe position at a time, we obtain the test results for all positions. This procedure can be done effectively since the genomic locations where the test presents uncertainty, and therefore, needs to be computed, are only a subset of all genome positions. The locations are dependent on the positions where the relevant DNA-probes are situated. More precisely, for a given window *w*, the test needs only to be performed for three positions: a window centered on the location *l *of the DNA-probe itself, and two windows centered at *l *- *w *and *l *+ *w*, i.e. centered at the end points of the first window. Consequently, tests are done for the three windows [*l *- 2*w*, *l*], [*l *- *w*, *l *+ *w*] and [*l*, *l *+ 2*w*] around the relevant DNA-probe. In total 3*k *tests are performed, where *k *is the number of relevant probes. This solution is computationally fast and allows a feasible multiple testing correction while providing the coverage of all genome positions relevant to the test. A Bonferroni correction for multiple testing is applied by multiplying the p-value of each test by the number of tests performed (3*k*). Note that the Bonferroni correction is a rather conservative correction, since the windows of observation of different DNA-probe may not be independent.

In order to identify the regions of aberration, we interpolate the corrected p-values of the hypergeometric test using the maximum value; i.e. given two successive locations with corrected p-value *a *and *b*, the base-pairs positioned between those locations are assigned the maximum of *a *and *b*. The base-pairs of the genome where the corrected p-value is smaller than 0.05 are considered significantly enriched for genomic aberrations. This step is repeated for different window sizes in order to detect both small and large aberrations. An illustrative result is shown in Figure 1 (Step 2). On the vertical axis are the different window sizes, the blue lines along the genome (the horizontal axis) show the regions judged significant by the algorithm.

STEP 3. The regions of aberration are identified based on a consensus between the results of the different window sizes. As illustrated in Figure 1 (Step 3), the number of window sizes for which a location is judged significant by the hyper-geometric tests are shown on the vertical axis. The "relevant" regions are the locations judged significant by at least *s *window sizes (the result for *s *= 9 is depicted by the red curve in Figure 1, Step 3). The researcher can decide to accept relevant regions as those in which any of the window sizes showed a significance, or may be more strict and demand the significance across several scales. The regions of chromosomal aberration are provided as output.

#### Complexity and scalability issues

Our real datasets are BAC aCGH, with ~ 3000 DNA-probes. The complexity of the SIRAC algorithm is 1) O
 MathType@MTEF@5@5@+=feaafiart1ev1aaatCvAUfKttLearuWrP9MDH5MBPbIqV92AaeXatLxBI9gBaebbnrfifHhDYfgasaacH8akY=wiFfYdH8Gipec8Eeeu0xXdbba9frFj0=OqFfea0dXdd9vqai=hGuQ8kuc9pgc9s8qqaq=dirpe0xb9q8qiLsFr0=vr0=vr0dc8meaabaqaciaacaGaaeqabaqabeGadaaakeaat0uy0HwzTfgDPnwy1egaryqtHrhAL1wy0L2yHvdaiqaacqWFoe=taaa@383C@(*N log*(*N*)), where *N *is the total number of elements (DNA-probes) in the array (since the SAM analysis has to be performed for each single probe), and 2) O
 MathType@MTEF@5@5@+=feaafiart1ev1aaatCvAUfKttLearuWrP9MDH5MBPbIqV92AaeXatLxBI9gBaebbnrfifHhDYfgasaacH8akY=wiFfYdH8Gipec8Eeeu0xXdbba9frFj0=OqFfea0dXdd9vqai=hGuQ8kuc9pgc9s8qqaq=dirpe0xb9q8qiLsFr0=vr0=vr0dc8meaabaqaciaacaGaaeqabaqabeGadaaakeaat0uy0HwzTfgDPnwy1egaryqtHrhAL1wy0L2yHvdaiqaacqWFoe=taaa@383C@(*k*), where *k *is the number of relevant elements selected by SAM (since the hypergeometric test is applied three times per relevant probe). Therefore, SIRAC can be used also with higher resolution aCGH, such as the cDNA or the oligo arrays. To give an indication of the time demands we have evaluated the run time of SIRAC on our computer server (an Intel Xeon 2.33 GHz with 8 G of memory). The run time for the NKI dataset with 2952 DNA-probes, and 692 relevant DNA-probes was 50 seconds; while SIRAC took 401 seconds to run on a cDNA array dataset with 30601 DNA-probes, 2532 of which were judged relevant by the SAM analysis.

## Experimental results

### Set-up

We illustrate our algorithm on an artificial dataset, described in the following Section and apply our method to two breast cancer datasets. The first dataset (*NKI*) is composed by 67 patients and 3219 BAC clones (DNA-probes). The samples are a selected series of the 295 breast cancer samples described in [28], and the BAC platform is discussed in [29]. The second dataset (*Fridlyand*) contains 67 samples and 2464 BAC clones, as described in [15].

In our proposed algorithm there are a few choices that the researcher has to make. A first important decision concerns the number of relevant DNA-probes. We choose to be conservative and require that the selected DNA-probes have a false discovery rate smaller than 0.005. This ensures that we include a very small fraction of false positive DNA-probes in further steps. Another parameter is the range of window sizes that are used to probe the genome. Since the average space between the clones is 1 Megabase (Mb), the minimum window of observation is set to 1 Mb. The maximum window size is fixed to 24 Mb because this is roughly half the length of the shortest chromosome. In this way, we enforce that the largest window does not always cover both the p and q arm of the chromosome.

## Results

### Artificial dataset

The artificial dataset is created using the clone distribution of the 207 clones of Chromosome 1 on the *NKI *array. The amplitude of the DNA-probes is drawn from a normal distribution with zero mean and unit variance N
 MathType@MTEF@5@5@+=feaafiart1ev1aaatCvAUfKttLearuWrP9MDH5MBPbIqV92AaeXatLxBI9gBaebbnrfifHhDYfgasaacH8akY=wiFfYdH8Gipec8Eeeu0xXdbba9frFj0=OqFfea0dXdd9vqai=hGuQ8kuc9pgc9s8qqaq=dirpe0xb9q8qiLsFr0=vr0=vr0dc8meaabaqaciaacaGaaeqabaqabeGadaaakeaat0uy0HwzTfgDPnwy1egaryqtHrhAL1wy0L2yHvdaiqaacqWFneVtaaa@383A@(0, 1). We chose to have two classes with 35 samples each. In Class 1 we simulate an amplification of amplitude *m *spanning *u *DNA-probes situated between positions *l*_*s *_and *l*_*e*_. The remaining DNA-probes have an average amplitude of zero. For Class 2 all samples have an average amplitude of zero for all DNA-probes. Zero mean, unit variance Gaussian noise is added to all samples across all DNA-probes. More formally, for a DNA-probe *p *at position *l *in a sample of class 1 the following holds:

p(l)={N(m,1),ls<l<leN(0,1),otherwise.
 MathType@MTEF@5@5@+=feaafiart1ev1aaatCvAUfKttLearuWrP9MDH5MBPbIqV92AaeXatLxBI9gBaebbnrfifHhDYfgasaacH8akY=wiFfYdH8Gipec8Eeeu0xXdbba9frFj0=OqFfea0dXdd9vqai=hGuQ8kuc9pgc9s8qqaq=dirpe0xb9q8qiLsFr0=vr0=vr0dc8meaabaqaciaacaGaaeqabaqabeGadaaakeaacqWGWbaCcqGGOaakcqWGSbaBcqGGPaqkcqGH9aqpdaGabeqaauaabaqaciaaaeaat0uy0HwzTfgDPnwy1egaryqtHrhAL1wy0L2yHvdaiqaacqWFneVtcqGGOaakcqWGTbqBcqGGSaalcqaIXaqmcqGGPaqkcqGGSaalaeaacqWGSbaBdaWgaaWcbaGaem4CamhabeaakiabgYda8iabdYgaSjabgYda8iabdYgaSnaaBaaaleaacqWGLbqzaeqaaaGcbaGae8xdX7KaeiikaGIaeGimaaJaeiilaWIaeGymaeJaeiykaKIaeiilaWcabaGaee4Ba8MaeeiDaqNaeeiAaGMaeeyzauMaeeOCaiNaee4DaCNaeeyAaKMaee4CamNaeeyzauMaeiOla4caaaGaay5Eaaaaaa@627C@

**Figure 2:**
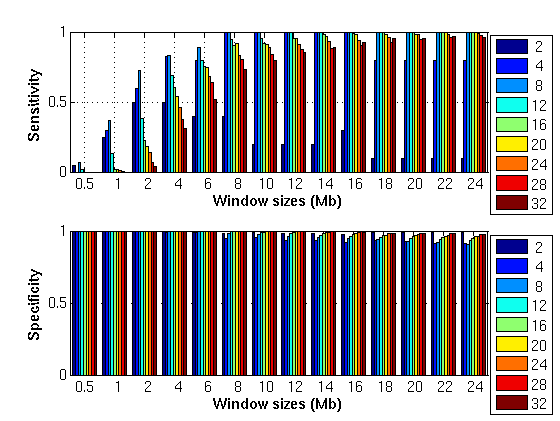
**Artificial dataset, sensitivity and specificity across different amplification lengths**. Sensitivity and specificity of the different window sizes for 10 instantiations of the artificial dataset with amplification amplitude *m *= 0.8. For each plot, on the horizontal axis are the distinct window sizes used to detect the amplification. The different color bars show the results for the individual amplification lengths *u *adopted.

The samples in the other class are all drawn from the normal distribution N
 MathType@MTEF@5@5@+=feaafiart1ev1aaatCvAUfKttLearuWrP9MDH5MBPbIqV92AaeXatLxBI9gBaebbnrfifHhDYfgasaacH8akY=wiFfYdH8Gipec8Eeeu0xXdbba9frFj0=OqFfea0dXdd9vqai=hGuQ8kuc9pgc9s8qqaq=dirpe0xb9q8qiLsFr0=vr0=vr0dc8meaabaqaciaacaGaaeqabaqabeGadaaakeaat0uy0HwzTfgDPnwy1egaryqtHrhAL1wy0L2yHvdaiqaacqWFneVtaaa@383A@(0, 1). The artificial dataset provides us with a ground truth which allows us to investigate the sensitivity and specificity of the algorithm and the effects of different window sizes. We applied our algorithm to amplifications with a range of amplitudes (*m *∈ {0.2, 0.4, 0.6, 0.8, 1}) and widths (*u *∈ {2, 4, 8, 12, 16, 20, 24, 28, 32} megabases (Mb)). Given the region of amplification found by the algorithm, the DNA-probes located in this region that also belong to the interval between positions *l*_*s *_and *l*_*e *_are defined as true positive, while the DNA-probes outside the interval are denoted false positives. Similarly, for the DNA-probes outside the region of amplification found by the algorithm, true negatives are the DNA-probes outside the interval between positions *l*_*s *_and *l*_*e*_, while false negative are the DNA-probes included in this interval. In general, the same trend for specificity and sensitivity as a function of *m *is observed. Figure 2 shows the average sensitivity and specificity for 10 different instantiations of the artificial dataset with the amplitude of the amplification *m *= 0.8. On the horizontal axis are the different window sizes used to detect the amplification. The different color bars show the results for the different amplification lengths, *u*, adopted. In the upper plot the sensitivity is shown. Let us focus on the amplification of length 2 Mb (dark blue bar). It can be seen that the maximum sensitivity is reached for window sizes of length 2 and 4 while the sensitivity decreases for larger window sizes. Similarly the amplification of length 16 Mb (green bar) is detected with the maximum sensitivity of 1 by a window size 18 Mb. Consequently, smaller window sizes detect small amplifications better, while larger window sizes more accurately reveal the larger amplifications. This behavior highlights the benefits of using window sizes of different lengths, to detect both large and small chromosomal aberrations. As expected, the specificity is maximal for small window sizes and decreases when larger window sizes are used. This behavior is due to the fact that wider window sizes include a larger number of false positives DNA-probes than the smaller windows sizes.

**Figure 3:**
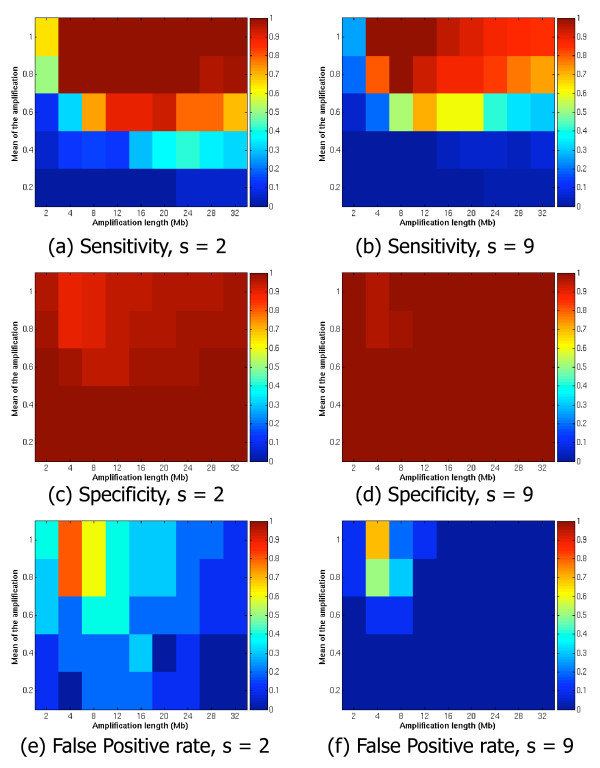
**Artificial dataset, sensitivity and specificity for *s *∈ {2, 9}**. Sensitivity, specificity and False Positive Rate (FPR) for two values of the parameter *s*, i.e. *s *∈ {2, 9}. For each plot, on the horizontal axis are the different amplification lengths *u*, used, and on the vertical axis are the different amplitudes of the amplification *m*. The colors code the value of the sensitivity, specificity and FPR from 0 to 1.

In our algorithm, we combine the different window sizes in order to obtain a unique region of amplification, by setting the parameter *s*. A location is amplified if it is judged amplified in *s *window sizes. We also investigated the effect of the parameter *s*. The top four plots of Figure 3 illustrates the sensitivity and specificity for two values of the parameter *s*, i.e. *s *∈ {2, 9}. We choose *s *= 2 as a loose constraint, while the more strict value of *s *= 9 requires the consensus of two-thirds of the window sizes. For each plot, the horizontal axis depicts the different amplification lengths, *u *used, and the vertical axis the amplitudes of the amplification, *m*. The colors code the value of the sensitivity and specificity from 0 to 1. The small amplification of *m *= 0.2 is very difficult to detect, therefore the sensitivity is very low regardless of the length of the amplification (bottom row of blue squares in Figure 3(a)). When the amplification amplitude increases, the sensitivity rises as well. If *s *= 9 fewer extremely large and small aberrations are not detected compared to *s *= 2, in other words, the sensitivity is lower when *s *= 9 compared to *s *= 2. However, at the same time, the specificity increases (Figure 3(d)).

In order to evaluate the control of the error rate, we computed the False Positive Rate (FPR), which is defined as FPFP+TP
 MathType@MTEF@5@5@+=feaafiart1ev1aaatCvAUfKttLearuWrP9MDH5MBPbIqV92AaeXatLxBI9gBaebbnrfifHhDYfgasaacH8akY=wiFfYdH8Gipec8Eeeu0xXdbba9frFj0=OqFfea0dXdd9vqai=hGuQ8kuc9pgc9s8qqaq=dirpe0xb9q8qiLsFr0=vr0=vr0dc8meaabaqaciaacaGaaeqabaqabeGadaaakeaadaWcaaqaaiabdAeagjabdcfaqbqaaiabdAeagjabdcfaqjabgUcaRiabdsfaujabdcfaqbaaaaa@3474@, with FP representing the number of False Positives and TP the number of True Positives. Figure 3(e) and 3(F) shows the FPR for *s *= 2 and *s *= 9 discretized into 10 equal sized intervals of size 0.1. We can observe that when *s *= 9 the FDR is mostly below 0.1, while the control of the FDR is not so strict when *s *= 2. However, the improved control of the FDR is achieved at the cost of the sensitivity. In the following experiments with real data, we choose to use the less stringent constrain of *s *= 2 to maximize the sensitivity. A further prioritization of the DNA-probes in a region can take into account the "strength" of the amplification. For example, the list of DNA-probes may be prioritized according to the number of window sizes in which each DNA-probe is judged aberrated. In this way, the strong aberrations can be differentiated from the weak ones.

### The NKI dataset

Sorlie and Perou [30-32] introduced the distinction of breast cancer into five different subtypes (Basal, ERBB2, Luminal A, Luminal B, Normal-like) based on the gene expression of the so called *intrinsic genes*. These genes were selected as the genes that had significantly greater variation in expression between different tumors than between paired samples of the same tumor. Using these genes, the profile of a centroid was obtained for each subtype. These centroids, in combination with the gene expression of 295 breast tumors [28] were employed to assign each sample in the *NKI *set to one of the subtypes based on its correlation with the centroid profiles across the intrinsic genes. In the *NKI *data, 21 out of 67 samples were labeled as Basal, 10 as ERBB2, 21 as Luminal A, 12 as Luminal B and 3 as Normal-like.

Recently, Bergamaschi *et al. *[33] studied the genomic aberrations of the different subtypes on a aCGH dataset. We applied our method to the *NKI *dataset and compare our findings to the results of Bergamaschi *et al. *[33]. More specifically, we applied the SIRAC algorithm four times, each time analyzing one subtype against the rest. The Normal-like subtype was not considered in this analysis due to the small number of samples.

**Figure 4:**
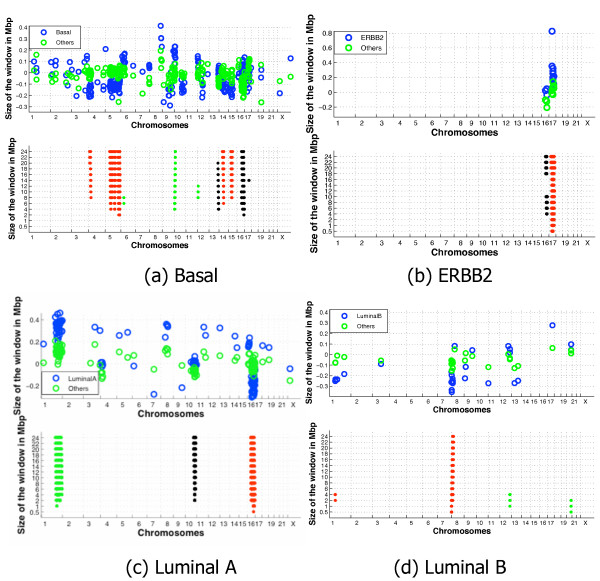
**NKI dataset, SIRAC results of Step 1 and 2 for the different cancer subtypes**. Results of Step 1 and 2 of the SIRAC algorithm for the four different subtypes in the *NKI *dataset. In the top panel of each figure the relevant DNA-probes detected by the SAM analysis are displayed on their genomic location (horizontal axis). For each relevant DNA-probe two circles are plotted at its location, representing the median of the class of interest (Basal, ERBB2, Luminal A or Luminal B) and the median of the remaining samples. In the bottom plot, the regions identified by the algorithm as significantly aberrated are shown for all genomic locations (horizontal axis) with a line for each window width used (vertical axis). The length of the line indicates the region on the genome that is significantly enriched with relevant DNA-probes. The color indicated whether the aberration is an amplification (green) or a deletion (red) in the class of interest, or an aberration in the other samples (gray).

Figure 4 shows the results of Step 1 and 2 of the SIRAC algorithm for the four different subtypes in the *NKI *breast cancer dataset. In the top plot of each figure the relevant DNA-probes detected by the SAM analysis are displayed. For each relevant DNA-probe two circles are plotted at its location on the genome, representing the median of the class of interest (Basal, ERBB2, Luminal A or Luminal B) and the median of the remaining samples. From these plots it is visible how some locations are significantly more densely populated by relevant DNA-probes than others. The lower plot of each subtype highlights the regions of aberration detected by SIRAC, color coded with the direction of the aberration. Green is used for an amplification, red for a deletion. Since our algorithm also highlights regions where the aberrations are not in the samples of the class of interest, when this occurs the region is depicted in gray, e.g. Chromosome 16 for the ERBB2 subtype.

**Figure 5:**
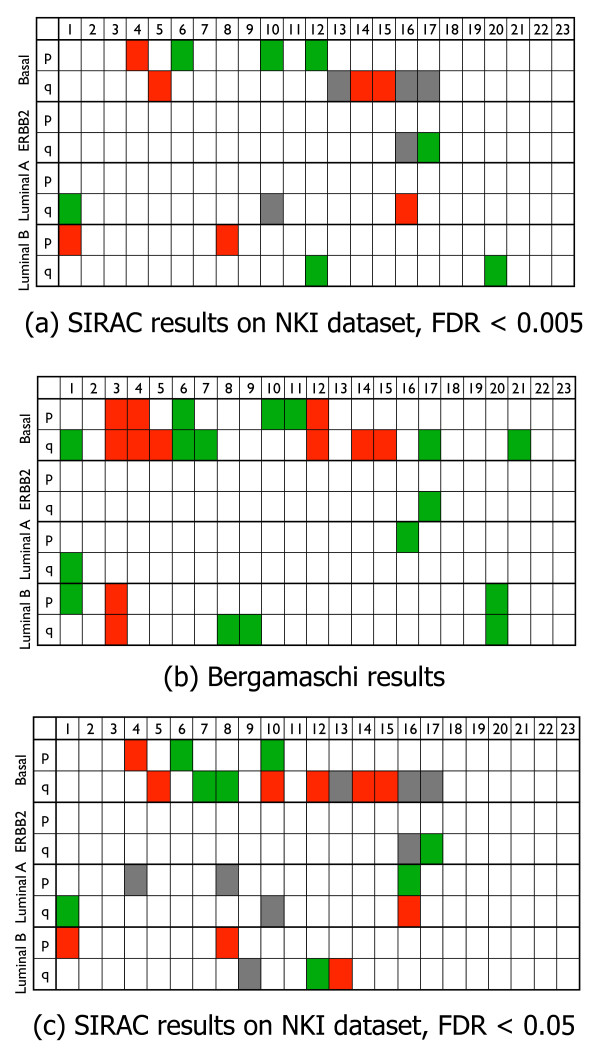
**NKI dataset, summary tables results and comparison with Bergamaschi et al**. Summary of the aberrations per chromosome arm for the four different subtypes (Basal, ERBB2, Luminal A or Luminal B). The numbers in the top of the tables denotes the chromosomes. A arm is indicated with a red color when a significant region is found on that arm that shows a deletion of the DNA-probes of interest. Similarly, green indicates amplification. The gray boxes indicate that the aberration was not present in the class of interest but in the rest of the samples. The top and the bottom tables show the aberrations found with the SIRAC algorithm on the *NKI *dataset for two different values of the FDR, i.e. FDR < 0.005 and FDR < 0.05 respectively. The middle table presents the results of Bergamaschi *et al. *[33] on their breast cancer dataset.

Figure 5(a) summarizes the aberrations found on the p or q chromosomal arms of the different subtypes when *s *= 2. The same color coding used in the lower plots of Figure 4 is applied to the chromosome arms, i.e. red specifies a deletion, green an amplification, and gray indicates that the aberration was not in the class of interest. Note that the resolution of SIRAC is neither restricted to chromosome arms nor to cytobands. The representation per chromosomal arm given in Figure 5 is adopted only for the sake of conciseness. The Basal subtype is associated with the largest number of aberrations, with deletions on Chromosomes 4, 5, 14 and 15, and amplifications on Chromosomes 6, 10 and 12. The ERBB2 subtype has only an amplification on the q arm of Chromosome 17, covering the genomic position where the ERBB2 gene is located. This is a known aberration, and the results suggest that this is the only aberration that differentiates this subtype from the other samples. The fact that this known aberration is found, also serves as a positive control for the SIRAC algorithm. The Luminal A subtype is characterized by a strong amplification on Chromosome 1 and a deletion on Chromosome 16. The Luminal B has less pronounce aberrations on Chromosomes 1, 8, 12 and 20.

We compared our findings with the conclusions of Bergamaschi *et al. *[33] that also searched for aberrations associated with subtypes on a *different *aCGH dataset. They first used the CLAC algorithm [14] to determine per sample the chromosomal gains and losses, then discretized the information per cytoband. Finally they use the SAM analysis to identify the aberrations correlated with the class labels. The aberrations found by them are summarized in Figure 5(b). In the Basal subtype, 6 of the 7 aberrations found by applying SIRAC to the *NKI *dataset are also in their list. The ERBB2 subtype only has the amplification on Chromosome 17, as in our findings. In the Luminal A subtype the strong amplification on Chromosome 1 is present while the one on the p arm of Chromosome 16 only reaches significance for an FDR = 0.05. In fact, as it is visible from Figure 4(c) , on the q arm of this chromosome many relevant DNA-probes show a deletion, while fewer DNA-probes on the p arm, although present, are not significant. In the Luminal B subtype, one of the three regions found by us is also present in Bergamaschi *et al. *[33] results.

Some of the differences between our results obtained on the *NKI *dataset and Bergamaschi results can be explained by the fact that our algorithm targets only the aberrations specific for a given class when compared to the rest of the samples. Therefore, we don't have the same aberrations for two subtypes. This is, for example, the case for the amplification on Chromosome 17 that is present both in the Basal and ERBB2 subtype for Bergamaschi *et al. *[33] while it is only a feature of the ERBB2 subtype in our results. Similarly, the amplification on the q arm of Chromosome 1 is a strong aberration only in the Luminal A subtype in the *NKI *dataset, while Bergamaschi *et al. *[33] reported it for both the Luminal A and the Basal subtypes. Another aspect to take into account is that we choose an FDR < 0.005 for the identification of the relevant DNA-probes by the SAM analysis. This rather strict value limits the number of false positives, and enables us to highlight the stronger aberrations. We repeated the experiments with a less strict constraint, i.e. using a FDR smaller than 0.05 or 0.1. The results for the FDR < 0.05 are shown in Figure 5(c). Four more aberrations were detected in the Basal, two of which are present in Bergamaschi *et al. *[33] (the amplification on Chromosome 7, and the deletion on the q arm of Chromosome 12). The ERBB2 still shows only the amplification on Chromosome 17. In the Luminal A subtype we detected one more amplification on the p arm of Chromosome 16, in agreement with the results of Bergamaschi *et al. *[33]. On the other hand, we find a few more aberrations for the Luminal B subtype, but these did not match the findings of Bergamaschi *et al. *[33].

Overall, given the differences in the datasets and in the methodology used, we can see striking similarities in the subtype characterization of the cancer. Especially the Basal, the ERBB2 and the Luminal A subtypes seem better defined, while the Luminal B type, seems rather weak, and we advocate that a better definition of this subtype needs to be established.

As stated earlier, we simply chose to represent the detected aberrations in terms of chromosome arms in order to ease the comparison with Bergamaschi *et al. *[33]. However, such a representation does not highlight a very useful feature of the SIRAC algorithm: the scale space. The scale space allows evaluation of aberrations at different genomic resolutions, and the number of scales across which an aberration remains significant can also be employed to judge the importance of a region, for a fixed SAM-FDR. By employing this feature, one can zoom in on potentially interesting regions, where the aberration has a larger average amplitude, and is of medium length (see Figure 1 (Step 3)). When increasing the number of scales (s) across which an aberration should be significant, the number of DNA-probes in significant regions across the genome is typically reduced strongly. More specifically if, for the *NKI *dataset, s is changed from s = 2 to s = 9, the number of DNA-probes in significant regions decrease from 174 to 56 for the Basal subtype (68% reduction), 76 to 31 for ERBB2 (59% reduction), 135 to 86 (36% reduction) for Luminal A and 33 to 7 (79% reduction) for Luminal B. Therefore, if only copy number is employed to identify putative regions (genes), the scale space analysis provides a powerful tool to reduce the list of genes putatively involved in the studied process.

### The Fridlyand dataset

Recently, Fridlyand *et al. *[15] analyzed the aberrations of 67 breast cancer samples. First they smoothed each sample using Circular binary segmentation [34], and defined chromosomal aberrations per sample. Based on the clustering of the smoothed data they identified three subtypes, i.e. the *1q16q*, the *Complex *and the *Mixed amplifier *subtypes. The *1q16q *subtype is named after the only copy number aberrations detected, i.e. a gain on 1*q *and a loss on 16*q*. The *Complex *subtype is characterized by many low level copy number alterations, mainly ER negative tumors, and worse outcome than the others subtypes. The *Mixed amplifier *subtype tumors were both ER positive and ER negative and did show several aberrations. They analyzed the aberration frequency in each subtype in order to find patterns of chromosomal changes across samples.

**Figure 6:**
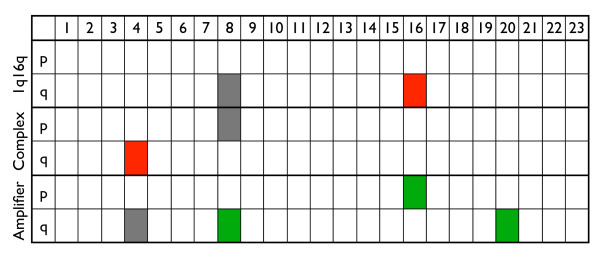
**Fridlyand dataset, summary tables results**. Summary of the aberrations per chromosome arms for the *Fridlyand *dataset [15]. The deletions are depicted in red, and the amplification in green, the gray boxes indicates that the aberration was significant not in the class of interest but in the rest of the samples.

We applied our algorithm to their data, analyzing each subtype against the remaining samples. Figure 6 summarizes our findings. We identified a loss on the q arms of Chromosomes 16 and 4 for the *1q16q *and the *Complex *subtypes respectively, and the amplifications on Chromosomes 8, 16 and 20 for the *Mixed amplifier *subtype. The comparison with the conclusions of Fridlyand *et al. *[15] is not straightforward, since their goal was not to identify aberrations specific for one class. Their results consist in a frequency plot for each subtype of the copy number changes more frequently associated with it. More specifically, they show the frequency of the clone aberrations present in more than 50% of the samples of one subtype and in less than 30% of the samples in the other subtypes. This illustration is not clearly pointing out the differences between subtypes, since often a percentage of the same aberration is present in two or more subtypes. However, our findings show correspondences with the results of Fridlyand *et al. *[15]. They define the class *1q16q *as exhibiting an amplification on Chromosome 1 and a deletion on Chromosome 16. We only detect the deletion on Chromosome 16. We think that the aberration on Chromosome 1, which is not detected by our algorithm, may be not specific for this class. From the data it is apparent that this amplification is present in all samples, i.e. not specific for the *1q16q *subtype. Other aberrations detected by our algorithm reflect a pattern in the frequency plot of Fridlyand *et al. *[15], such as for the deletion in 4*q *of the *Complex *subtype and the amplification in 8*q *of the *Mixed amplifier *subtype. In other cases, such as the amplifications on Chromosomes 16 and 20 in the *Mixed amplifier *class, our findings are not reflected in the frequency plot of Fridlyand *et al. *[15]. In conclusion, the results of SIRAC and Fridlyand *et al. *[15] exhibit partial overlap. The advantage of our algorithm is that it better highlights the differences between subtypes and clearly points out the specific chromosomal aberrations.

## Discussion and conclusion

We have presented a method to identify aberrant chromosomal regions that are specific for the problem under study. Our emphasis is not on the identification per sample of a chromosomal gain or loss, but we strive to evaluate what makes two classes different from each other, and what are the aberrations that distinguish them. We also want to limit the number of preprocessing steps, in order to reduce the set of inevitable parameters to be tuned. This motivated us to avoid the characterization per sample of the DNA-probes being amplified or deleted, which is instead the necessary input data for the STAC algorithm [18] and the approach followed by Fridlyand *et al. *[15]. We chose to use the raw data as input and assumed that a DNA-probe amplified/deleted in one class and not in the other is selected as significant by the SAM analysis. Of course the researcher has to choose the appropriate false discovery rate. This decision influences the number of DNA-probes preselected as relevant. This is an important starting point of our algorithm. We opted for a low false discovery rate for all the problems analyzed. The different number of relevant DNA-probes selected in the distinct cases already gave us an indication of the number and the strength of the chromosomal aberrations. For example in the *NKI *dataset the largest number of relevant DNA-probes was present in the Basal subtype, while the ERBB2 class was associated with only a few DNA-probes mainly on Chromosome 17.

Our algorithm is designed to identify the copy number alterations in the aCGH data. The core of the algorithm resides in the identification of the regions of chromosomal aberration. We assumed that an aberration involves more than a single DNA-probe. Therefore, we tested in a systematic manner the candidate regions, i.e. the locations in the vicinity of the DNA-probes identified by the SAM analysis. The use of different window sizes allows us to detect different lengths of copy number changes and not to miss aberrations in regions sparsely covered by the aCGH probes. Since for the samples in the *NKI *data also the expression is available, we tested if similar results could be obtained by applying our algorithm to the expression directly, as Furge *et al. *[19] did. However, the assumption that an over/under expression should involve more than a single gene here does not hold anymore. Even if a region is amplified, not all genes may be active and, therefore, differentially expressed with respect to the reference. Moreover, while in the aCGH data the only cause of aberration resides in the copy number variation, the variance in the expression is due to multiple factors. In general, we observed in our expression dataset that the relevant genes selected by the SAM analysis were scattered across the genome and, therefore, no clear regions of significance were identified. This result further indicates that the detection of genomic aberration using gene expression datasets should be performed with caution, and results should always be validated with other tests, such as FISH or PCR, if not with genomic copy number data itself.

Instead, the expression data can be used to perform a post-processing step on the algorithm applied to the aCGH data. Once the aberrated regions have been identified, the expression data allows for a further analysis of the genes present in these regions. For example, the genes can be prioritized according to the correlation between the expression and the aCGH data, or according to the ability of each gene to distinguish between the classes of interest. This is especially relevant since we expect that, for instance, not all genes in a region of aberration will be active, some may be silent and not contributing to the mechanism of cancer. A selection can be done based on this additional information source, resulting in a smaller list of potentially interesting genes to be further analyzed. The benefits of the use of the expression data are exemplified by the ERBB2 subtype in the *NKI *dataset. The genes present in the amplified region of Chromosome 17 were ranked according to the product of the p-value of the t-test (computed on the gene expression and class labels) and the p-value of the correlation between the expression of each gene and its closest DNA-probe. The top two genes are the ERBB2 gene itself and the GRB7, i.e. the growth factor receptor-bound protein 7. This is expected since the ERBB2 subtype is characterized by the amplification of the ERBB2 gene, and the GRB7 is found to be over-expressed and co-amplified with the ERBB2 gene [22,35,36]. Therefore, a combined approach of SIRAC and the use of gene expression is a powerful additional tool in the search for marker genes.

In the SIRAC algorithm we first detect associations of single probes with the class label, and then search for regions that are enriched for class label associated probes. This is advantageous especially when working with tumor samples. The heterogeneity of the tumors may lead to signals for the aberrations smaller than the ones expected if the sample cells were homogeneous. Therefore, amplifications/deletions with small absolute values may be of interest as well, especially when they discriminate the classes of interest. Several authors (e.g. Saramaki *et al. *[37], Fridlyand and Chin *et al. *[15,38], and Nymark *et al. *[39]) have recently pointed out that even low-level copy number aberrations may have significant effects on the gene-expression and, therefore, on the cell functioning and tumor development.

The error rate control of SIRAC is performed in two different steps. First the null-hypothesis being constructed during the permutation steps of the SAM procedure, second, the Bonferroni correction for multiple testing applied to the p-values of the hypergeometric test. The artificial experiment illustrates how the dependencies between these two steps may lead to an anti-conservative control of the error rate. The choice of the parameter *s*, which combines the outcomes of different window sizes, plays an important role. The artificial experiments suggests that the stricter the value, e.g. *s *= 9, the better the control of the error rate. However, this is achieved at the expenses of the sensitivity. Therefore, less conservative choices, e.g. *s *= 2, may be used. In this case, the p-values of the hypergeometric test need to be interpreted with caution. The SIRAC algorithm, however, provides useful details, such as the number of window sizes in which each DNA-probe was judge significant, that can be used to further prioritize the regions. Moreover, if the expression data is available, further validation of the aberrations may be performed by investigating the correlation with the expression of the genes in the identified region.

In conclusion, we focused on the identification of the chromosomal aberrations that discriminate between the classes of interest and proposed a robust algorithm for the evaluation of their significance. Our algorithm does not require preprocessing of the data such as discretization or smoothing, and uses a limited number of parameters. Our findings on the two breast cancer datasets are in agreement with previous studies, and better highlight the dissimilarities between the classes of interest.

## Appendix

**Algorithm 1 **SIRAC: Supervised Identification of Relevant Aberration in aCGH datasets

1: **Input: **dataset *D*, label set **y**, SAM parameters: *d *for the desired false discovery rate and number of iterations *I*; vector **W **with half the sizes of observation windows; threshold *t *for the hyper-geometric distribution; minimum number of windows sizes *s *for which the location is judged significant.

2: Apply the SAM analysis with the given parameters *d *and *I *to the labeled dataset *D*, **y**. A vector **J **stores the indexes of the relevant DNA-probes obtained.

3: Initialize variables: *P *= *ones*(|**W**|, 3|**J**|), stores the p-value of the test; *POS *= *zeros*(|**W**|, 3|**J**|) stores the location where the test is applied.

4: *∀ w ∈ ***W **(for all window sizes)

5:    Initialize: bon = 0; (count the number of tests performed)

6:    ∀*j *∈ **J **(for all relevant DNA-probes)

7:       Determine position of the window centers **C **= [*l*^*j *^- *w*, *l*^*j*^, + *w*] around the DNA-probe, with *l*^*j *^the position of the *j*th DNA-probe.

8:       If C
 MathType@MTEF@5@5@+=feaafiart1ev1aaatCvAUfKttLearuWrP9MDH5MBPbIqV92AaeXatLxBI9gBaebbnrfifHhDYfgasaacH8akY=wiFfYdH8Gipec8Eeeu0xXdbba9frFj0=OqFfea0dXdd9vqai=hGuQ8kuc9pgc9s8qqaq=dirpe0xb9q8qiLsFr0=vr0=vr0dc8meaabaqaciaacaGaaeqabaqabeGadaaakeaat0uy0HwzTfgDPnwy1egaryqtHrhAL1wy0L2yHvdaiqaacqWFce=qaaa@3824@*h*(*l*^*j *^- *w*) = C
 MathType@MTEF@5@5@+=feaafiart1ev1aaatCvAUfKttLearuWrP9MDH5MBPbIqV92AaeXatLxBI9gBaebbnrfifHhDYfgasaacH8akY=wiFfYdH8Gipec8Eeeu0xXdbba9frFj0=OqFfea0dXdd9vqai=hGuQ8kuc9pgc9s8qqaq=dirpe0xb9q8qiLsFr0=vr0=vr0dc8meaabaqaciaacaGaaeqabaqabeGadaaakeaat0uy0HwzTfgDPnwy1egaryqtHrhAL1wy0L2yHvdaiqaacqWFce=qaaa@3824@*h*(*l*^*j*^) = C
 MathType@MTEF@5@5@+=feaafiart1ev1aaatCvAUfKttLearuWrP9MDH5MBPbIqV92AaeXatLxBI9gBaebbnrfifHhDYfgasaacH8akY=wiFfYdH8Gipec8Eeeu0xXdbba9frFj0=OqFfea0dXdd9vqai=hGuQ8kuc9pgc9s8qqaq=dirpe0xb9q8qiLsFr0=vr0=vr0dc8meaabaqaciaacaGaaeqabaqabeGadaaakeaat0uy0HwzTfgDPnwy1egaryqtHrhAL1wy0L2yHvdaiqaacqWFce=qaaa@3824@*h*(*l*^*j *^+ *w*), with C
 MathType@MTEF@5@5@+=feaafiart1ev1aaatCvAUfKttLearuWrP9MDH5MBPbIqV92AaeXatLxBI9gBaebbnrfifHhDYfgasaacH8akY=wiFfYdH8Gipec8Eeeu0xXdbba9frFj0=OqFfea0dXdd9vqai=hGuQ8kuc9pgc9s8qqaq=dirpe0xb9q8qiLsFr0=vr0=vr0dc8meaabaqaciaacaGaaeqabaqabeGadaaakeaat0uy0HwzTfgDPnwy1egaryqtHrhAL1wy0L2yHvdaiqaacqWFce=qaaa@3824@*h *a function that assigns the chromosome number of the corresponding base pair location

9:       Then

10:          Initialize: **H **= *ones*(1, 3), (stores the test value for the triplet position in **C**)

11:          ∀ *c *∈ **C **(for all window positions)

12:             h=∑i=0xℋ(i|M,k,N)
 MathType@MTEF@5@5@+=feaafiart1ev1aaatCvAUfKttLearuWrP9MDH5MBPbIqV92AaeXatLxBI9gBaebbnrfifHhDYfgasaacH8akY=wiFfYdH8Gipec8Eeeu0xXdbba9frFj0=OqFfea0dXdd9vqai=hGuQ8kuc9pgc9s8qqaq=dirpe0xb9q8qiLsFr0=vr0=vr0dc8meaabaqaciaacaGaaeqabaqabeGadaaakeaacqWGObaAcqGH9aqpdaaeWaqaamrtHrhAL1wy0L2yHvtyaeHbnfgDOvwBHrxAJfwnaGabaiab=TqiijabcIcaOiabdMgaPjabcYha8jabd2eanjabcYcaSiabdUgaRjabcYcaSiabd6eaojabcMcaPaWcbaGaemyAaKMaeyypa0JaeGimaadabaGaemiEaGhaniabggHiLdaaaa@4A7F@ with:

13:                *x *= number of relevant DNA-probes in the window [*c *- *w*, *c *+ *w*],

14:                *M *= number of DNA-probes in the dataset *D*,

15:                *k *= number of relevant DNA-probes in the dataset *D*,

16:                *N *= number of DNA-probes in the window [*c *- *w*, *c *+ *w*].

17:             *H*^*c *^= 1 - *h*;

18:             bon = bon+1; (update the counter)

19:          End

20:          *P*^*wj *^= **H**; (*P*^*wj *^is the p-value on row *w *and probe triplet *j*);

21:          *POS*^*wj *^= **C**; (*POS*^*wj *^stores the triplet window location);

22:    *P*^*w *^= *P*^*w *^× *bon*; (Bonferroni correction)

23: ∀*l *∈ **G **(all positions in the genome):

24:    Fl=∑wNlw
 MathType@MTEF@5@5@+=feaafiart1ev1aaatCvAUfKttLearuWrP9MDH5MBPbIqV92AaeXatLxBI9gBaebbnrfifHhDYfgasaacH8akY=wiFfYdH8Gipec8Eeeu0xXdbba9frFj0=OqFfea0dXdd9vqai=hGuQ8kuc9pgc9s8qqaq=dirpe0xb9q8qiLsFr0=vr0=vr0dc8meaabaqaciaacaGaaeqabaqabeGadaaakeaacqWGgbGrdaWgaaWcbaGaemiBaWgabeaakiabg2da9maaqababaGaemOta40aa0baaSqaaiabdYgaSbqaaiabdEha3baaaeaacqWG3bWDaeqaniabggHiLdaaaa@37D7@, (*F*_*l *_= number of window sized where the test is above the threshold t), with:

25:       Nlw={1,if ∃ja|lja≤l≤lja+1&max(Pwya,Pwya+1)≤t),0,otherwise.
 MathType@MTEF@5@5@+=feaafiart1ev1aaatCvAUfKttLearuWrP9MDH5MBPbIqV92AaeXatLxBI9gBaebbnrfifHhDYfgasaacH8akY=wiFfYdH8Gipec8Eeeu0xXdbba9frFj0=OqFfea0dXdd9vqai=hGuQ8kuc9pgc9s8qqaq=dirpe0xb9q8qiLsFr0=vr0=vr0dc8meaabaqaciaacaGaaeqabaqabeGadaaakeaacqWGobGtdaqhaaWcbaGaemiBaWgabaGaem4DaChaaOGaeyypa0ZaaiqabeaafaqaaeGacaaabaGaeGymaeJaeiilaWcabaGaeeyAaKMaeeOzayMaeeiiaaIaey4aIqIaemOAaO2aaSbaaSqaaiabdggaHbqabaGccqGG8baFcqWGSbaBdaahaaWcbeqaaiabdQgaQnaaBaaameaacqWGHbqyaeqaaaaakiabgsMiJkabdYgaSjabgsMiJkabdYgaSnaaCaaaleqabaGaemOAaO2aaSbaaWqaaiabdggaHjabgUcaRiabigdaXaqabaaaaOGaeiOjayIaemyBa0MaemyyaeMaemiEaGNaeiikaGIaemiuaa1aaWbaaSqabeaacqWG3bWDcqWG5bqEdaWgaaadbaGaemyyaegabeaaaaGccqGGSaalcqWGqbaudaahaaWcbeqaaiabdEha3jabdMha5naaBaaameaacqWGHbqycqGHRaWkcqaIXaqmaeqaaaaakiabcMcaPiabgsMiJkabdsha0jabcMcaPiabcYcaSaqaaiabicdaWiabcYcaSaqaaiabb+gaVjabbsha0jabbIgaOjabbwgaLjabbkhaYjabbEha3jabbMgaPjabbohaZjabbwgaLjabc6caUaaaaiaawUhaaaaa@75F5@

26: **Output: **all locations with *F*_*l *_≤ *s*.

## Availability and requirements

Project name: SIRAC

Project home page: 

Operating system(s): Platform independent

Programming language: Matlab

## Authors' contributions

CL, HMH, MJvdV, MJTR and LFAW designed the experiments and analyzed the results; CL carried out the analysis; HMH generated the *NKI *dataset; EHvB and PMN set up the BAC platform and software employed to profile and pre-process the *NKI *dataset; all authors read and approved the final manuscript.
